# Inducing a sense of worthiness in patients: the basis of patient-centered palliative care for cancer patients in Iran

**DOI:** 10.1186/s12904-021-00732-3

**Published:** 2021-03-02

**Authors:** Mir Hossein Aghaei, Zohreh Vanaki, Eesa Mohammadi

**Affiliations:** Faculty of Medical Sciences, Tarbiat Modares University, Tehran, Iran

**Keywords:** Patient centered care, Palliative care and cancer

## Abstract

**Background:**

Patient-centered care is one of the main components in providing palliative care for cancer patients. This issue has been the subject of numerous studies and practices in nursing for many years. Few studies, however, have explored the perception of nurses about patient-centered treatments. This study aimed at exploring the perception of care-providers about offering patient-centered care to cancer patients.

**Method:**

For attaining that aim, 18 care-providers were purposefully selected for an interview which allowed the researchers to explore the enriched experiences of these participants about offering patient-centered palliative care to cancer patients. After transcribing the recorded data, analysis was carried out based on Graneheim and Lundman’s method of content analysis. The research was qualitative in nature and conducted in 2019 in Iran.

**Results:**

From content analysis, 3 main categories; considering patient’s spirituality, maintaining patient’s dignity during care, and reducing patient’s suffering, were found. The essence of these categories reflect on the care-providers’ consideration and effort in “*inducing a sense of worthiness”* in patients by providing patient-centered care. These categories also reflect on the perspective of care-providers about nurse- patient relationship for providing high qualified palliative care.

**Conclusion:**

For providing patient-centered care inducing a sense of worthiness in patients, is the most fundamental component in providing palliative care to cancer patients. Therefore, by considering the structures and settings where the care is to be provided in the healthcare system, it is possible to direct the necessary educational, research, and administrative programs related to inducing a sense of worthiness in patients towards providing a more effective palliative care**.**

## Background

Today, healthcare systems have proposed palliative care as in interdisciplinary procedure which focuses on improving the quality of patient’s life to provide effective care to cancer patients [1–3]. Providing care and supporting patients and their families through the various stages of life-threatening diseases like cancer, from the stage of diagnosis to the final day of a patient’s life and even after the patient’s death, is one of the goals of palliative care [4, 5]. To achieve these goals, the palliative care approach requires a framework and some principles, and patient-centered care is one of the basic principles which makes effective care possible [6–8]. In the patient-centered approach, the care-providers try to encourage patients to perform self-care by giving them specific information and instructions [9]. In healthcare systems, providing patient-centered care is a qualifying factor. Additionally, patient-centered care is a necessary element required for improving the care system and winning public trust, which in turn reduces the average time of patient’s hospitalization, increases patient satisfaction, and cuts hospital costs [10–12]. Providing patient-centered care has the potential to improve the health of individuals and societies by involving patients who have received sufficient information and motivation to control their disease, in their treatment program [9]. However, having patients motivated enough to control their disease is one of the many challenges of providing palliative care to cancer patients; and, the way care-providers motivate these patients reflects on the main concept or intention of providing patient-centered care.

The results of different studies [13–17] on patient-centered care have reflected on different features of such treatment such as communicating with patient, expressing sympathy, mutual decision-making, concentrating on patient’s preferences, observing the patient as a whole rather than in parts, and providing organized care. These features mainly manifest themselves in the general context of moral communication, and the morality itself is the dominant element. Although patient-centered care has been the focus of a number of studies and its significance has been emphasized by care-providers, it still faces various challenges when it comes to its application [18]. On the other hand, while the requirements of palliative care has developed gradually (for instance, the number of patients receiving the care in different societies is increasing), the patient-centered approach has also been modified [6]. Additionally, the structure of providing palliative care is one of the basic aspects that explains why patient-centered approach is required in health care systems [6]. Understanding the features and nature of patient-centered care is necessary especially when it is carried out in different fields of care and in various cultural settings [19, 20]. The care-providers’ perception and experiences with this subject plays a significant role here.

Understanding the nature of a process like patient-centered care has helped to comprehend why and how relevant challenges occur and it can also help develop or even improve the process itself to make it more practical. Therefore, as the quality of care increases, more goals are achieved in the care-providing system. Hence, the necessity of providing patient-centered care becomes an important motivation to investigate the experiences and perception of care-providers; and, as there are few studies that have been conducted on this issue in Iran, the present study aimed at exploring the experiences of care-providers with offering patient-centered palliative care to cancer patients in Iran.

## Method

### Research design and questions

The aim of this study was to explore healthcare providers’ perception about patient-centered care in providing palliative care for cancer patients. The research question was mainly concerned about the nature of care providing, in palliative care, and to the best of the researchers’ knowledge, no studies have been conducted with this particular focus in Iran. For data analysis, conventional content analysis approach was used to better understand the nature of patient-centered care in providing palliative care to cancer patients [21].

### Participants

Participants were healthcare providers with rich experiences in providing palliative care for cancer patients. Healthcare providers in this study included nurses, psychologists, clergymen, physicians, and patients’ family members. Purposive sampling was used and in selecting the participants their ability to well express their experiences was considered in addition to the participants’ contentment to collaborate. In Tehran; Firoozgar Hospital, the Palliative Care Department and Aalla Relief Service Charity Center were the selected setting of this research which all provided palliative care. Sampling was performed by maintaining maximum diversity in selecting participants based on sex, number of years of providing care, and different professions in providing palliative care. Before carrying out the research, each participant was informed about the purpose of the study as well as the method of interview. Eventually, 18 interviews were held with healthcare providers and patients’ family members; (specifically, 8 nurses, 2 home caregivers, 2 physicians, 2 psychologists, 1 social worker, 1 Spiritual counselor, and 2 patients’ family members.**)**

### Data gathering

Non-structured deep face-to-face interviews were held individually with the participants. Each interview took between 25 to 70 min. The place and time of interviews were arranged based on the participant’s preference and agreement in order to ensure that they are comfortable during the interview. Participants were informed about the purpose of the study prior to conducting the interviews, and after collecting their demographic information, the main questions of the interview were asked openly. Based on the participants’ answers, the next part of the interview was carried out to find answers to more explorative questions to learn more about the participants’ experiences regarding the concerned issue. All interviews were carried out in Persian and recorded by a digital voice recorder. Examples of the interview questions are provided were:

“Please describe your experience with the patients for whom you have performed patient-centered care”; and “please describe your experience with the caring services that you provide your patients”.

Interviews were carried out until data saturation was reached and no other themes or new categories were found in the interviews.

### Data analysis

Graneheim and Lundman’s qualitative content analysis approach was used for data analysis [22, 23]. After conducting each interview, the recorded information were transcribed word-for-word and the text was reviewed by the researchers a number of times to ensure a comprehensive understanding of the content. Then, semantic units were identified in the texts of the interviews, and primary codes were extracted. Codes were compared with each other and based on their similarities, their differences, and contents, they were placed in categories and subcategories. Then, relevant subcategories were placed in one category and eventually those categories which shared a common content, made a single theme. For example, Table 1 shows how the category of “Focusing on reducing patient’s suffering” was reached.

**Table 1:** The process of reaching “Focus on reducing patient’s Suffering” category

Category	subcategory	code	Participants’ experience
Focusing on reducing patient’s Suffering	Reducing Patient’s physical Suffering and pain	Using prescribed medicine to reduce pain	“If our patient is in pain, we try to inject the medicine that has been prescribed for him/her as soon as possible to reduce his/her pain”
		Reducing the pain caused by physical side-effects	“We try to overcome the physical side-effects that appear in patients, one-by-one, and try to reduce the amount of pain they feel”
	Reducing patient’s mental Suffering and pain	Trying to reduce patients’ fear of future	“They fear the future that has not yet come and we do our best so that our patients remain positive enough to reduce their fear and anxiety”
		Considering reducing patient’s concern and anxiety	“We try to reduce patients’ concern and anxiety by being beside them”
	Reducing imposed pressure from care on patient’s family	Keeping an eye on the family to reduce patient’s pain	“We try to keep an eye on patients’ families because when patients observe that their families are calm, they become more tolerant and hurt less”
		Reducing patient’s family problems while reducing patient’s pain	“The pressure that the patient’s family endures when providing care, also exercises pain on the patient and we try to reduce this pressure on families”

### Research rigor

The four criteria of transferability, dependability, credibility, and conformability were used to increase research rigor [24]. The credibility was established by use of prolonged engagement, as well as member- and peer-checking techniques. Also, the results were given to two palliative care nurses who did not participate in the study. They were asked to compare the results with their own work experiences. To perform peer check (in addition to the expert colleagues who were involved in the study), two qualitative researchers approved the extracted primary codes and categories. Transferability was attained through detailed data description which allowed the readers to evaluate the accuracy of the research and match the findings with their own contexts.

## Results

From data analysis, at first 215 main codes, 10 subcategories and 3 categories (“Maintaining patient’s dignity during care”, “Considering patient’s spiritual needs” and “Focusing on reducing patient’s pain”) were attained. And finally one theme “Inducing a sense of worthiness in the patient” was emerged inference (Table 2).

**Table 2:** The concept of “inducing a sense of worthiness in the patient” along with its categories and subcategories

Theme	Categories	Subcategories
**Inducing a sense of worthiness in the patient**	Considering patient’s spiritual needs	• Providing a spiritual environment for the patient • Considering patient’s spiritual concerns • Respecting patient’s spiritual beliefs
	Maintaining patient’s dignity during care	• Considering patient’s right of decision-making • Respecting patient’s privacy • Considering patient’s uniqueness • Building a respectful relation
	Focusing on reducing patient’s suffering	• Reducing patient’s physical suffering and pain • Reducing patient’s mental suffering and pain • Reducing imposed pressure on patient’s family

### Inducing a sense of worthiness in the patient

Worthiness is an inner potential that influences the way individuals perceive themselves. This certain perspective is also influenced by external sources; for example, in providing palliative care, care-providers are one of the most significant external sources that affect patients’ sense of worthiness. Based on data, this was represented in the present study as the following theme: “inducing a sense of worthiness in the patient”. This concept reflects on care-providers’ special attention to patients’ values. Participants’ experiences showed that whatever was valuable to patients, significantly affected the palliative care providing system; therefore, the care providing team had to consider and respect it as well. Paying special attention to these values and respecting them implies how crucial this issue is in patient-centered palliative care. Inducing a sense of worthiness in patients when providing palliative care is determined by the following three features: “considering patient’s spiritual needs”, “maintaining patient’s dignity when providing care”, and “focusing on reducing patient’s suffering”.

#### “Considering patient’s spiritual needs”

Spirituality is a personal experience which allows one to explore the nature of the world. It forms one’s profound values, and their concept of life. By analyzing the gathered data in this study, it was shown that considering a spiritual space for patients is one way to respect their spirituality. The spiritual atmosphere allows the patient to connect with a supernatural source of tranquility. Care-providers can enhance the comfort of patients by providing a spiritual place, the Qur’an, or even playing an audio of its reading for them. In this regard, a participant stated:

“We had a patient who felt hopeless and null. I brought him the Qur’an many times and told him to read one or two of its verses when he felt like it” (P1: Nurse)

Another participant stated:

“When patients feel that they don’t have a future anymore, I tell them that if they’d like, there is a prayer room here and there is a Qur’an in it, you can just go there and return. They might not even pray, but they feel that the place is safe for them to talk to God or just talk alone to themselves” (P12: Nurse)

Paying attention to the concerns of cancer patients is one of the manifestations of caring for patients’ spirituality. In this regard, care-providers considered and cared for patients’ spiritual requests and queries, and helped them to address their concerns. A participant stated the following about his/her experience with a patient who asked a question about death:

“If they’d directly ask a question about death, I’d answer them. Some may say that this is life and that they are afraid of death; here, we would take the conversation a little further to talk about this issue and help the patient to understand that death is something that happens to everyone”. (P13: Home care-giver)

Data showed that care-providers pay attention to patients’ spiritual needs, beliefs, and thoughts, which is proof that they consider patients’ spirituality when providing patient care. Care-providers have emphasized that even if patients have beliefs that are different or even opposing to their own, it does not impact their consideration and respect. In fact, they believe that understanding differences can even enhance their consideration of patients’ spiritual needs. In this regard, a participant shares the following experience:

“Some patients have special beliefs like they might tie a green piece of cloth around their wrest. My colleagues and I don’t attempt to take it off their wrests because even if we don’t believe in these stuff the patient does and so we’d say it would be better to just leave it there”. (P3: Nurse)

Another participant shares:

“When it comes to patients we don’t try to impose our own beliefs on them, or to convince them that their thought is wrong or that some other thought is right. We allow them to talk and we just direct them” (P5: Home Caregiver)

#### “Maintaining patient’s dignity during care”

One of the main features here is respecting the patient’s right to decide about the provided care and services. Ethically, it is the patient’s right to make the final decision and s/he can accept or refuse a treatment. However, occasionally at times when for example the patient is dubious about whether to be hospitalized, or whether to follow through with chemotherapy, the care-providing team gets involved and explains the condition and different settings to the patient in order to help them make a final decision with their families by giving them sufficient information. A participant stated:

“We explain and clarify issues like following through with chemotherapy and becoming hospitalized to patients and help them to make their decisions with their families”. (P4: Palliative care physician).

Participants’ experiences show that respecting patient’s privacy is also a feature of maintaining patient’s dignity during care. Palliative care-providers have been paying close attention to patients’ satisfaction and willingness when communicating with them. In this regard, a spiritual counselor stated:

“Our relation with the patient also depends on the patient's satisfaction and willingness. The patient somehow shows whether s/he is willing to get a consult or not. Now, this can be reflected in their looks, behavior, like when some may roll their eyes or some don’t even feel like talking, some pretend to be asleep, and well, we consider all these different situations” (P14: spiritual consultant).

Also, another nurse believed that care-providers should not make a fuss about communicating with patients or providing their care-related education especially when patients are reluctant:

“Some patients build a fence around themselves and don’t feel like doing anything, and here we try to respect their privacy. For example, we don’t try to make a big deal about communicating or providing care-related education” (P2: Nurse).

Another feature of this category is considering that each patient is a unique case. Patient’s beliefs or thoughts are just one aspect of their uniqueness; and in this study, care-providers have emphasized the significance of considering this issue when providing palliative care. A psychology consultant explained:

“When consulting patients, we try to receive them as a whole, considering all their thoughts and beliefs, because these are unique in every patient and we don’t try to impose our own thoughts on them … ” (P11: Psychologist)

Also, social workers have pointed to the difference among patients’ characters and concerns, as well as the different ways of responding to them:

“The patient alone is important to us with all of his/her thoughts and concerns. All these help us to apply the best method to address their problem” (P15: social worker).

Findings showed that in patient-based care, developing a respectful communication with the patient is an important feature. Palliative care providers try to communicate with patients by using kind words and treating patients warmly and respectfully. A participant stated:

“Most times when we want to develop a good relation with our patient and want the patient to feel that s/he is respected, we use kind and respectful words like “dear father” or “dear mother”.” (P2: Nurse)

Another participant also shared:

“We try to pay more attention to them and treat them with more respect and kindness so that they’d feel comfortable and valued” (P10: Nurse)

#### “Focusing on reducing patient’s suffering”

This is another feature that reflects on valuing patients during care. It stands for the care-providing team’s effort in reducing patient’s pain. Cancer patients endure different kinds of pain throughout their treatment such as physical, psychological and mental pain, and the care-providing team pays attention to all of them when providing palliative care. Participants’ experiences showed that cancer patients suffer from constant and progressive pain, and the care-providing team tries its best to reduce it. A nurse shared:

“We inject palliative medicine and we try to do everything we can for the patient because usually the patient is spending his/her final days or months and we believe that these last days shouldn’t be so painful”. (P18: Nurse)

For reducing the patient’s pain and suffering, the palliative care physician emphasizes his/her concern about preventing the side-effects of using higher doses of narcotics, as this results in a harm of its own kind:

“Many of our patients have access to low-quality drugs, and they use high doses of them while we try to turn those high-doses of low-quality drugs into lower doses of qualified industrial medication so that the side-effects are minimized and the patient gets to endure lesser pain as well” (P17: Palliative care physician).

Care-providers have discussed reducing not only the physical pain of cancer patients, but also their psychological and mental pain. The treatment process for many of these patients is lengthy which becomes at times quite exhaustive and may put the patient in distress. A participant shared a relative experience:

“One of our patients had shaved his head and even wanted to shave her eyebrows as well because she believed that after taking chemo she would suffer to watch all her hair fall out one-by-one, so she decided to get rid of her hair and for that matter to get rid of her anxiety. In such cases, we try to communicate with patients to reduce their psychological and mental distress”. (P6: Nurse)

Participant number 3 stated:

“One of the most important issues I have observed in these patients is that they develop a bad feeling when they learn that they have been diagnosed with cancer. This mentally disturbs patients making them anxious, and we try to reduce this anxiety when we are providing their care” (P3: Nurse)

Data showed that one of the factors that exercises pain and suffering on cancer patients is the pressure of care-giving that their family members endure and at times may pass on to them. Cancer patients, as a result, may feel that they are a burden while their family members may find themselves drowning in the patient’s problems and stressful care. Here, care-providers support the patient’s family members and try to reduce the pressure imposed on them while in turn they help reduce patient’s pain this way as well. Participant 15 shared an experience:

“In our follow-up, the patient's family is also supported. For example, we had a patient here whose companion encountered a problem while he/she also was under the pressure of supporting and taking care of the patient. This also turned into the patient’s concern which we tried to address to reduce his/her pain” (P15: Social Worker).

Another participant shared the following:

“When we constantly have to be beside the patient, well, somehow we lose track of our own lives and this makes us anxious. I used to cry all the time at first, and the psychological team here held some counseling meetings for me to deal with my anxiety and also prevent it from being transferred to my patient which if it did, it would elevate his/her pain”(P8: patients’ family members)

## Discussion

The aim of the present study was to explore the perception of palliative care-providers about providing patient-centered care for cancer patients. In this study, care-providers attempted to induce a sense of worthiness in the patient in order to perform patient-centered care for them. Considering the condition that cancer patients go through, it is comprehendible that they have to endure a great deal of pain due to which the patient’s sense of worthiness towards him/herself may be deteriorated. Care-providers try to induce a sense of worthiness in cancer patients in order to help them believe that they are important and loved by helping them to develop a better perspective about themselves. To this end, care-providers have paid attention to issues like patient’s spirituality, dignity, and different types of pain. Regarding all three issues (which were defined in terms of separate categories of “respecting patient’s spirituality, maintaining patient’s dignity during care, and reducing patient’s pain”), the participants emphasized that all their effort was to help patients feel more worthy and valuable instead of feeling useless.

Sandsdalen et al. [25] conducted a systematic review on the significance of patient’s priorities and preferences in receiving palliative care. The theme of this study was obtaining a meaningful life by providing patient-centered care, for which the following were required: a care providing system, care providing staff, proper environment for care, and care organization. These prerequisites together help to provide patient-centered care to patients, and also help patients to experiences a meaningful life. Sandsdalen et.al’s study was in concordance with the present study. Since care-providers are the most crucial prerequisite for providing patient-centered care [26], the present study has also focused on this issue along with the induction of a sense of worthiness (the theme of this study) in patients, and the concept of having a meaningful life becomes a subcategory of feeling worthy. In other words, if a patient achieves a meaningful life it shows that his/her spirituality has been valued, and paying attention to the spirituality of patients while also inducing a sense of worthiness to patients, makes them more receptive to accepting palliative care. This issue has been emphasized from various perspectives in other studies [27–31] such as: paying attention to patient’s spirituality as a factor that could help improve patient’s quality of life, adapting to the evolving concerns, providing hope for the patient, and finding ways to help patients escape their spiritual crisis and improve their lives.

Patients receive palliative care in different cultures and societies; so, despite the commonality of human nature, they will differ in terms of their spirituality, culture and beliefs [32–34]. Therefore, different strategies will need to be applied to provide a more effective care for patients. The culture and faith of the people of Iran is Islamic, based on the Quran; as Muslims claim that after death, man is moved from this life to the everlasting world [35]. While the phenomenon of death and reflection is profoundly traumatic, in the culture and belief of Iranian society, holding the Quran overhead and patient beside it, reading the Quran and remembering the divine word induces inner peace in people who are life-threatening and close to death [36, 37] . It is often believed that all the pain that a human faces during his or her life is a divine challenge [37] and that having a sacred space for patients to learn about these values reinforces their sense of meaning. In addition, the teachings of Iranian people pay attention to the value of the patient at the time of death and after death; at the time of death, measures such as shutting the eyes and mouth of the patient, reciting prayers, maintaining a relaxed atmosphere and placing the patient’s feet in the direction of the Macca are done in order to respect the deceased, relatives and family of the patient [36, 37]. Despite the discrepancies in the specifics of various sects and traditions on the topic of faith, this problem is highlighted in different communities in order to give patients a sense of meaning. In the present study, participants noted that one aspect of paying attention to patients’ spirituality is respecting their beliefs; and by addressing patient’s spiritual needs, care-providers can induce a sense of worthiness in them. Ferrell et.al have investigated this issue in dealing with cancer patients, and have discussed how considering and respecting these patients’ beliefs is a strategy that helps evaluate patient’s spirituality [29].

The participants in this study believed that by providing a spiritual environment, they can support cancer patients’ spirituality which in turn would help patients to become much calmer. Phelps et al. showed that providing a spiritual environment for patients positively changes their attitude and makes them feel supported [38]. This feeling of being supported was introduced as the sense of worthiness in the present study. As also reported in Ragsdale and Zeighamy’s study, providing a spiritual environment for patients is one way to show that their spirituality is valued and respected; and it is also a strategy to address patients’ spiritual needs by helping them connect with Their divine source of tranquility or their inner self [39, 40]. Additionally, Zeighamy and Docherty believe that this is a spiritual need and it helps to develop more respect for patients’ spiritual beliefs, which allows care-providers try to help patients overcome their spiritual concerns, and it also helps patients to develop relations with others [40, 41].

Maintaining patient’s dignity is another important issue in inducing a sense of worthiness in patients. The participants of this study had mentioned this in their interviews and had explained in order to maintain patients’ dignity how they had applied different strategies such as: considering patient’s right to decide and choose, treating each patient as a unique case, respecting patient’s personal space and privacy, and developing a respectful relation with the patient. In a study conducted by Eklund et al. [42], all available review articles on patient-centered care were collected and analyzed, where the results showed that there are two concepts that underlie this method: patient-centeredness and person-centeredness which have their own sub-concepts: empathy, communication, respect, mutual decision-making and being unique. These concepts are in concordance with the concept of maintaining patient’s dignity (as proposed in the present study) although in different studies the concept of person-centeredness has been mentioned to be something beyond the concept of patient-centeredness whose aim is to achieve a meaningful life. It can be inferred that patient-centeredness or person-centeredness are conceptually derived from a common theme which is inducing a sense of worthiness in the patient, by which the patient is considered as a person - not just an individual who is receiving treatment - who has dignity and in whom care-providers try to induce a sense of worthiness. Maintaining human dignity as a moral value in palliative care, and in different studies, it is one of the important concepts of patient-centered care [43, 44].

Franck’s study showed that paying attention to individual’s intrinsic capacity and applying patient-centered palliative care can potentially help to give a new meaning to their life and develop a sense of well-being in the patient [45]. It is a human right to have the liberty to choose how to react when encountering different situations, and in the present context this stands for both the patient and care-providers. Yet, while the condition of some patients may worsen and they are left with no other option but to take the treatment, considering and respecting patients’ perspectives and decisions in choosing or refusing palliative care, is a significant role of the care-providing system and it is what makes it ethical. The results of this study were also in line with Janson’s which showed that the palliative care approach considers the patients as unique individuals when it respects their liberty and ability to make decisions about the care that they are receiving – although these decisions are made by the help of the patient’s family and care-provider [46].

Palliative care is associated with valuing morality, experiencing a body of emotions, and expressing those emotions. Guo and Pringle have stated in their studies that the most important moral value in providing palliative care is maintaining a person’s dignity, which is also an important concept in providing patient-centered care [43, 44]. The results of this study also showed that the central value in ethical care is a person’s dignity which is addressed when a patient is respected and a constructive correlation has been built between them and the care-providers that would also allow the induction of a sense of worthiness in the patients. This communication which is associated with respect, forms a reliable supporting relation between the patient receiving palliative care and the care-provider, and this will eventually help calm the patient [47]. Additionally, other studies have noted that when palliative care providers have a reliable and respectful relationship with their patients, they can understand patients’ stress and pain as well as that of their families, and hence they can support them better during the difficult times when they may encounter challenges during their treatment [48, 49]. Seccareccia et al. have also mentioned the significance of developing a respectful relation with the patient as a higher level of valuing them, and states that this will induce a sense of belong in the patients making them more receptive to their treatment [50]. Therefore, it can be inferred that developing a respectful relation with the patient is so crucial that makes the other aspects of care providing smoothly possible. Without such a relation, through which effective mutual communication is possible, the patient will not feel valued or respected and may even quit his/her palliative care. On the other hand, the socio-cultural setting within which the care is provided can also affect the applied strategies for developing a relation with patients and their families; for example, in an Iran, using terms like “Dear father” or “Dear Mother” are in fact effective as they hold positive connotations and transfer a sense of kindness, respect and closeness to the patient and their families, which facilitates the care-provider’s attempt to build a closer relation with the patient. Focusing on reducing patient’s suffering is another feature of inducing a sense of worthiness in cancer patients who are receiving palliative care. The present study explored this issue from various aspects of reducing patients’ physical, mental, and psychological pain. One of the significant ethical aspects of providing patient-centered care is focusing on patients’ pain and anxiety while comforting them. Belanger’s study is also relevant to this issue and considers nurses’ activities a caring response to patients’ needs [51].

In this study, focusing on reducing patient’s pain is one of the main aspects of paying attention to patient’s needs, and care-providers tried to reduce the pain by inducing a sense of worthiness in patients. The results of other studies have also considered the attempt to reduce patient’s physical, mental and psychological pain as a principle of paying attention to the patient’s needs; and to do so, they have used different strategies such as music therapy, yoga, and psychological support to reduce the patient’s pain [52–56]. In addition to emphasizing the consideration of patients’ physical and mental aspects in reducing patients’ pain, the results of this study have also highlighted the significance of reducing the pressure imposed on caregivers.

In concordance with the results of the present study, other studies [57, 58] have also expressed their concerns about the attention that patients who have to take palliative care should receive from their care-providers and family members. This was also a salient issue in this study which shows how a family member could be quite dependent on the entire family especially in an Iranian society. By reducing the pressure imposed on the families because of taking care of their patient, it is possible to calm both families and patients, which in turn would help patients to become more tolerant of their pain. Still, other studies [59–61] have reported that taking care of the patient’s family is one of the main considerations of palliative care, however, the findings of this study stated that reducing the family’s pain with the aim of reducing patient’s pain is a part of patient-centered care which enhances the sense of worthiness in the patient.

## Conclusion

Findings showed that patient-centered treatment is not merely considering the uniqueness of patients or attending to their needs, but in fact, it is a procedure in which the direction of treatment and its outcome is important – which is to induce a sense of worthiness in the patient. Findings are also promising in developing educational, administrative and research programs in the healthcare system, especially in countries which are still at the initial stages of organizing their palliative care. In this respect, patient-centeredness becomes a practical and directional concept in inducing a sense of worthiness in patients, which goes beyond assessing the services that are provided to merely address patients’ needs.

Developing the concept of inducing a sense of worthiness as a framework for research or a scale to assess patient-centered services, can be a step in providing quality care that would also bring patients’ satisfaction and inner serenity. Therefore, these results can be used to design a tool to evaluate or assess patient-centeredness in a treatment program. Additionally, the results of the present study can also be combined with palliative care quality assessment tools and be investigated as a significant aspect of caring, and it can be used as a method that has been proved to be effective as it considers patients’ values and induces a sense of worthiness in them (which are significant aspects of care-providing) .

In concordance with the results of the present study, it is necessary to pay attention to clinical, educational and research bases to ensure the performance of palliative care providers. In order to optimize patient-centeredness based on inducing a sense of worthiness in patients, providing training or educational programs for the professional care-providing team can be fruitful. These training programs can be in accordance with the concepts mentioned in the present study (i.e. respecting patients’ spirituality, maintaining patients’ dignity when providing care, and reducing patients’ pain) to expand patient-centered caring strategies. On the other hand, creating a proper structure for providing palliative care in different environments, especially hospitals and homes, and organizing these cares to form a value-centered trend in providing care and developing it can leave a great impact on its care and development.

## Data Availability

The datasets (original interviews) generated and/or analysed during the current study are not publicly available due the source of the data is the interviews conducted with participants, which in addition to maintaining confidentiality in the names and *details* of participants, the size of the interview file is also high, but they are available from the corresponding author on reasonable request.
